# Geospatial Distributions of Groundwater Quality in Gedaref State Using Geographic Information System (GIS) and Drinking Water Quality Index (DWQI)

**DOI:** 10.3390/ijerph16050731

**Published:** 2019-02-28

**Authors:** Basheer A. Elubid, Tao Huag, Ekhlas H. Ahmed, Jianfei Zhao, Khalid. M. Elhag, Waleed Abbass, Mohammed M. Babiker

**Affiliations:** 1 Department of Environmental Science and Engineering, Faculty of Geosciences and Environmental Engineering, Southwest Jiaotong University, high-tech zone, Chengdu 611756, China; basheer@my.swjtu.edu.cn (B.A.E); taohuang70@126.com (T.H.); zhaojianfei@my.swjtu.edu.cn (J.Z.); 2 Department of Hydrogeology, Faculty of Petroleum & Minerals, Al Neelain University, Khartoum 11121, Sudan; elubaid@yahoo.com (B.A.E.); elubbash@hotmail.com(W.A.); 3 School of Resources and Environment, University of Electronic Science and Technology of China, Chengdu 611756, China; 201714180103@std.uestc.edu.cn; 4 Key Laboratory of Digital Earth, Institute of Remote Sensing and Digital Earth, Chinese Academy of Sciences, No. 9 Dengzhuang South Road, Haidian District, Beijing 100094, China; khalidelhag@mails.ucas.ac.cn; 5 Water Environmental Sanitation Project (WES), Gedaref State Water Corporation, Gedaref 32214, Sudan; ahmed_geonuv@163.com

**Keywords:** aquifers, drinking water quality index DWQI, spatial distribution, piper diagram, interpolation methods

## Abstract

The observation of groundwater quality elements is essential for understanding the classification and distribution of drinking water. Geographic Information System (GIS) and remote sensing (RS), are intensive tools for the performance and analysis of spatial datum associated with groundwater sources control. In this study, groundwater quality parameters were observed in three different aquifers including: sandstone, alluvium and basalt. These aquifers are the primary source of national drinking water and partly for agricultural activity in El Faw, El Raha (Fw-Rh), El Qalabat and El Quresha (Qa-Qu) localities in the southern part of Gedaref State in eastern Sudan. The aquifers have been overworked intensively as the main source of indigenous water supply in the study area. The interpolation methods were used to demonstrate the facies pattern and Drinking Water Quality Index (DWQI) of the groundwater in the research area. The GIS interpolation tool was used to obtain the spatial distribution of groundwater quality parameters and DWQI in the area. Forty samples were assembled and investigated for the analysis of major cations and anions. The groundwater in this research is controlled by sodium and bicarbonate ions that defined the composition of the water type to be Na HCO_3_. However, from the plots of piper diagram; the samples result revealed (40%) Na-Mg-HCO_3_ and (35%) Na-HCO_3_ water types. The outcome of the analysis reveals that several groundwater samples have been found to be suitable for drinking purposes in Fa-Rh and Qa-Qu areas.

## 1. Introduction

Groundwater is a noble resource for water in arid and semiarid areas [1,2,3,4,5,6]. Accessibility to water is an important global goal whose effects are abundantly felt in developing countries. The benefit of understanding groundwater geochemistry is to ensure its good quality for drinking [7,8,9]. In arid and semi-arid areas, the potential use of groundwater for drinking and agricultural projects is threatened by the decline of water quality due to physical and anthropogenic characteristics. Evaluation of the geochemical status of groundwater is required to competently plan and control the groundwater resources [10]. The interaction between water and rocks has usually been studied to provide an understanding of the physical and chemical procedures controlling water chemistry [11,12,13]. Several factors control the groundwater geochemistry such as the type of rock forming the aquifer, the residence time of water in the hosted aquifer, the origin of the groundwater and the flow directions of groundwater [14,15].

The estimation of quality and the use of groundwater for different purposes are becoming more significant [16]. Thus, probes related to an understanding of the hydro-chemical aspects of the groundwater, geochemical processes and its development under natural water flowing manners, not only aids in the practical utilization and protection of this expensive resource but also aid in visualizing the changes in the groundwater environment [17,18].

Statistical analysis methods such as the correlation matrix, bivariate, and Hierarchal component analysis; produce a reliable alternative procedure for understanding and explaining the complex system of water quality with the capability of analyzing large amounts of data [19].

GIS and RS, are intensive tools for performance and analysis of spatial datum associated with groundwater sources control. Remote sensing data are essential in many geo-resources, such as mineral research, hydrogeology, and other geologic fields [20]. It is important in hydrogeological reconnaissance for understanding structural, geomorphological, and lithological features. The acquired RS information improves our knowledge of the hydrogeological conditions. Satellite images are universally applied for qualitative estimation of groundwater resources by investigating geological structures, geomorphic features, and their hydrological characteristics [21,22,23]. The spatial distribution maps were designed and integrated within ArcGIS v.10.5 software.

Drinking water quality index (DWQI) presents a single number to reveal the overall water quality at a particular position and time, based on various water quality factors. It is interpreted as a number that indicates the combined impact of several water quality parameters [19,24,25,26,27,28]. DWQI has been popularly utilized in water quality evaluations for both surface and sub-surface water, and it has represented an increasingly significant function in the water resource environment and management [29,30,31].

Shortage of drinking water in East Central Sudan especially in the basaltic terrain is a common problem. Most of the rural community depends on groundwater sources in their daily life for drinking purposes. This research aims to generate groundwater distribution maps and to evaluate water quality for drinking purposes in Qa-Qu and Fw-Rh areas in the southern part of Gedaref State in eastern Sudan. The area consists of one of the essential agricultural fields of Gedaref State, the El Rahad project which was developed as a mechanized project in Sudan in 1978 [32].

In this work, forty samples were observed and monitored from selected boreholes. Hence, an attempt of statistical analysis methods such as correlation matrix and Hierarchal component analysis were applied to determine the variation in hydro-chemical facies and understand the development of hydro-chemical processes. Moreover, adopting Piper and Durov diagrams by use of Aqua Chem. v.2014.2 software, to classify the groundwater facies and water types in the area. The world health organization (WHO) [33] standard has been used for correlations with the results of sample analysis to examine the permissible amount of water for drinking.

The analytical results achieved from the samples when plotted on Piper’s plot, explained that the alkalis (Na^+^, K^+^), appear considerably over the alkaline elements (Ca^2+^, Mg^2+^), and the weak acidic (HCO_3_^−^) appear considerably over strong acidic anions (Cl^−^ & SO_4_^−2^). Moreover, the Piper diagram matched 40% of the samples, under Na-Mg-HCO_3_ group and 35% under Na-HCO_3_ type. According to the plotting from the Durov diagram, most of the elements of water plotted within the HCO_3_·Na zone, except some other samples that fell in HCO_3_ Cl-Na, SO_4_·Cl·HCO_3_-Na, or HCO_3_·Cl-Na·Mg types. DWQI was calculated by adopting weighted arithmetical index methods considering thirteen water quality parameters (pH, TDS, Ca^+2^, Mg^+2^, Na^+^, K^+^, Fe^+2^, Cl^−^, HCO_3_^−^, SO_4_^−2^, F^−^, NO_3_^−^, and E.C) in order to assess the degree of groundwater contamination and suitability for drinking purposes.

For the better understanding of geological units in this project, the thin sections of rock samples have been generated. With this ability, the rock mineral contents have been determined much better. This study has great importance; due to the plan for obtaining drinking water from the groundwater sources to Fw-Rh and Qa-Qu localities. However, this investigation is helpful in understanding groundwater environments and its suitability for human uses, especially in arid and semi-arid regions.

## 2. Materials and Methods

### 2.1. Geology

**Figure 1:**
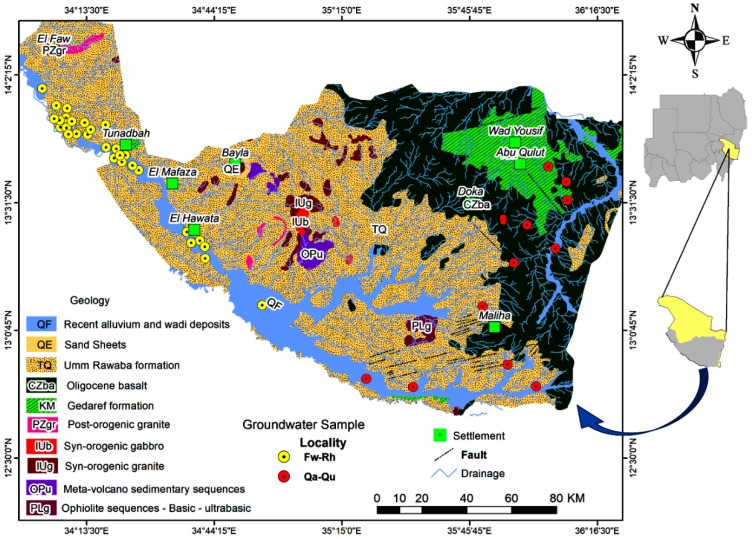
Location map and geological units of study area.

Geologically Figure 1; the lower Proterozoic rocks of the basement complex (Mainly Granitic Gneisses) [34], Syn-orogenic Granit and Syn-orogenic gabbro underlain the sandstone of Gedaref formation, Tertiary (Oligocene) basalt [35], Umm Rawaba formation, sand sheets and recent alluvium and wadi deposits. The groundwater of the area was taped at the sandstone of Gedaref formation sequence, alluvium soil, and fractures of the Oligocene basalt aquifers with depths ranging from 14 to 64 m.

### 2.2. Hydrogeological Setting

**Figure 2:**
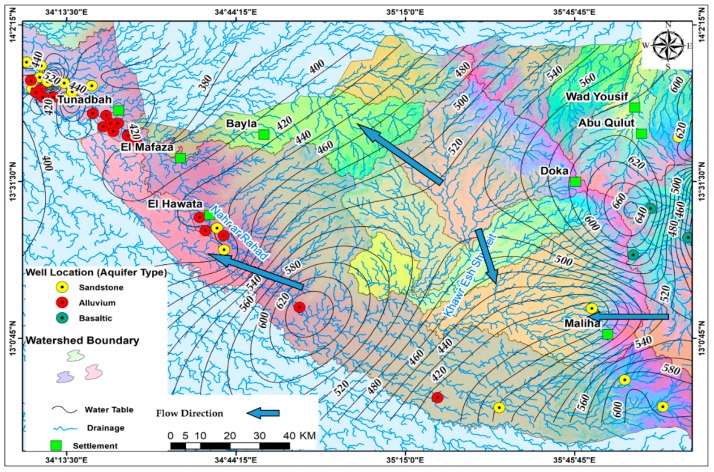
Hydrogeological map of the study area.

The groundwater was studied by using the data collected from forty boreholes drilled in Fw-Rh and Qa-Qu area as seen in Table 1. The hydrogeological characteristics of rock units were investigated, and aquifer systems were determined depending on field investigations and previous studies. Therefore, the hydrogeological map of the Fw-Rh and Qa-Qu area was settled adopting ArcGIS v.10.5 software, based on characteristics of the lithological units Figure 2. According to these evaluations, the aquifer types were described as sandstone, alluvium, and fracture basalt.

### 2.3. Spatial Interpolation and Groundwater Quality Mapping

Spatial interpolation is a procedure of predicting the value of attributes at unsampled sites from measurements made at point locations within the same area [36]. There are two main groupings of interpolation techniques: deterministic and geostatistical. Deterministic interpolation techniques create surfaces from measured points, based on either the extent of similarity (e.g., Inverse Distance Weighted) or the degree of smoothing (e.g., radial basis functions). Geostatistical interpolation techniques (e.g., kriging) utilize the statistical properties of the measured points.

In this study, we found that the Kriging (Ordinary and Simple) interpolation method is the most suitable method. Thus, the histograms and normal QQplots were plotted to examine the normality distribution of the observed data for each water quality element in both Fw-Rh and Qa-Qu localities.

### 2.4. Drinking Water Quality Index DWQI

DWQI has been determined based on the standards of drinking water quality as counseled by WHO. Therefore, thirteen chemical parameters (pH, TDS, Ca, Mg, Na, K, Cl, HCO_3_, F, NO_3_, Fe, and E.C.) were used for the calculation. To apply DWQI in the current study, the study area was divided into two parts, Fw-Rh, and Qa-Qu localities. The water quality parts were generated by a weighting factor and then formerly aggregated by using the simple mean calculations. To estimate the water quality in this project, the quality rating (Q_i_) for all elements was estimated through the following equation;

(1) $$Q_{i}=\left\{\left(V_{a}-V_{i}/V_{s}-V_{i}\right)\right\}*100$$

where, Q_i_ = Quality ranking of the element form a total number of water quality elements, V_a_ = Real amount of the water quality element taken from laboratory study, V_i_ = Ideal rate of the water quality element can be realized from the standard Tables. V_i_ for pH = 7 and for other elements it is equaling to zero. V_s_ standard = Value of WHO standard.

Then, the Relative weight (W_r_) was studied from inversed proportional of recommended standard (S_i_) for the corresponding parameter using the following expression;

(2) $$W_{r}=\frac{I}{S_{i}}$$

Here W_r_ = Relative (unit) weight for specific element; Si = Standard allowable amount for certain element; I = Proportionality constant.

Assuredly, the total DWQI was determined using the assemblage equations of the quality rating with the unit weight linearly as the following:

(3) $$\mathrm{DWQI}=\;\sum Q_{i}W_{r}/\;\sum W_{r}\#$$

where Qi = Quality rating; Wr = Relative weight.

In general, DWQI is determined for particular and intended uses of water. In this work, the DWQI was estimated for human consumption, and the maximum DWQI value for the drinking purposes was regarded as 100 scores.

**Figure 3:**
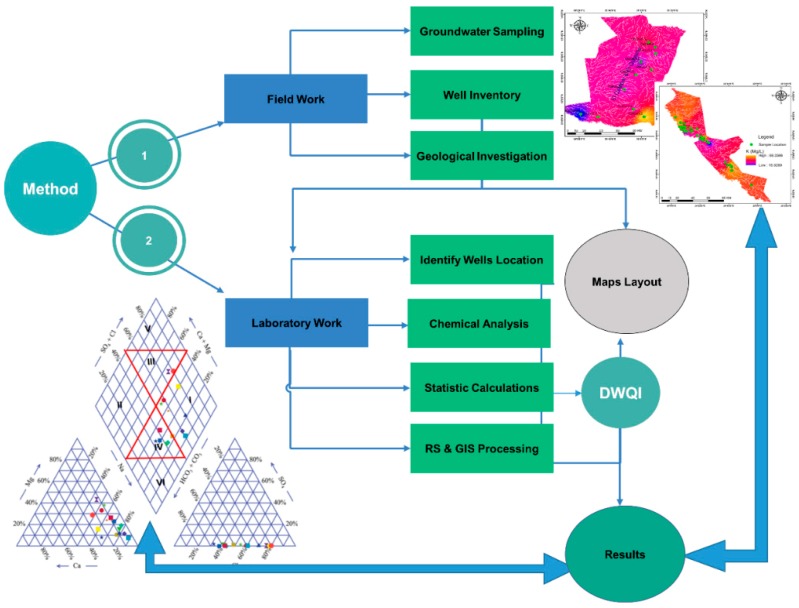
Methodology chart of study procedures.

The methodology ideas in this work have been done through several steps Figure 3.

## 3. Results

Several factors may control the groundwater geochemistry such as the type of rock forming the aquifer, residence time of water in the hosted aquifer, the origin of the groundwater and the flow directions of groundwater. Hydro-chemical properties of the groundwater of the area are shown in Table 2. The water pH ranges between 7.5 and 8.9, indicate an alkaline chemical reaction in both sandstone and basaltic aquifers. The electrical conductivity (E.C) varies from 345 to 3342 μS/cm.

### 3.1. Interpolation and Elements Distribution Maps

The quality of interpolation is described by the difference of the interpolated value from the true value. Thus, the Anderson-Darling test, which is an ECDF (empirical cumulative distribution function) based test, tests the prospect that the value of a parameter falls within a particular range of values (confidence level 95%). The data points are relatively close to the fitted normal distribution line. The p-value is greater than the significance level of 0.05. Subsequently, the scientist fails to reject the null hypothesis that the data follow a normal distribution.

**Figure 4:**
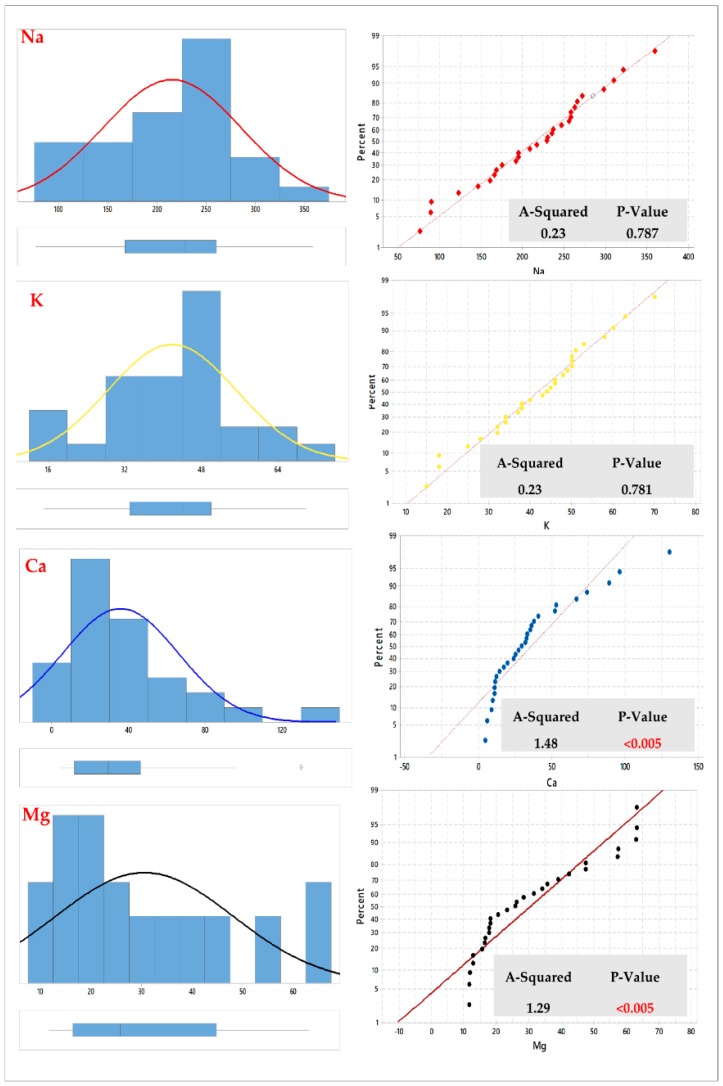
Graphical summary and probability plots of cations in Fw-Rh area.

**Figure 5:**
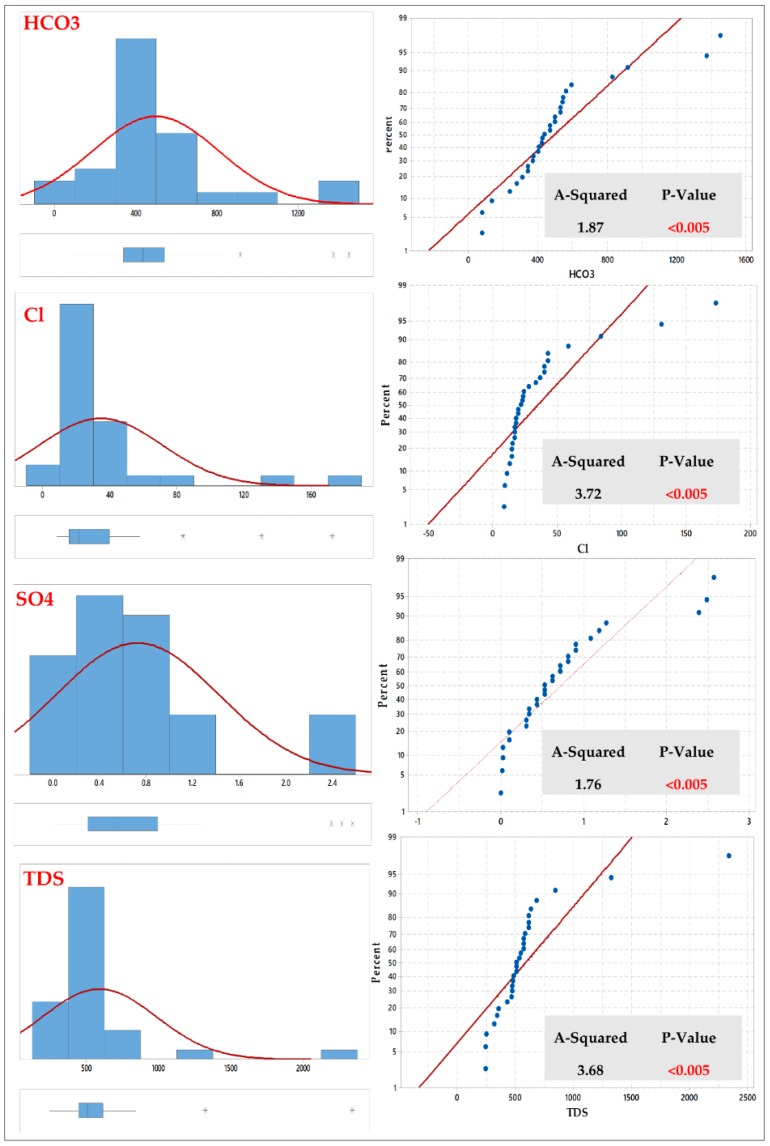
Graphical summary and probability plots of HCO_3_^−^, SO_4_^−2^, Cl^−^ & TDS in Fw-Rh area.

According to this test, in Fw-Rh area we found that the parameters (Na and K) showed a normal distribution when the other elements (Ca, Mg, HCO3, Cl, SO4 and TDS) showed a more or less abnormal distribution in Figure 4 and Figure 5.

The same test has been performed in (Qa-Qu) area, which showed that the (Mg and SO_4_) parameters reflected normal distribution while the other variables (Ca, K, Na, HCO_3_, Cl, and TDS) present non-normally distributions.

Generally, most of the collected elements in both Fw-Rh and Qa-Qu localities were skewed. However, the transformations (Log & BoxCox), have been used to make the data normally distributed and satisfy the assumption of equal variability for the data.

**Figure 6:**
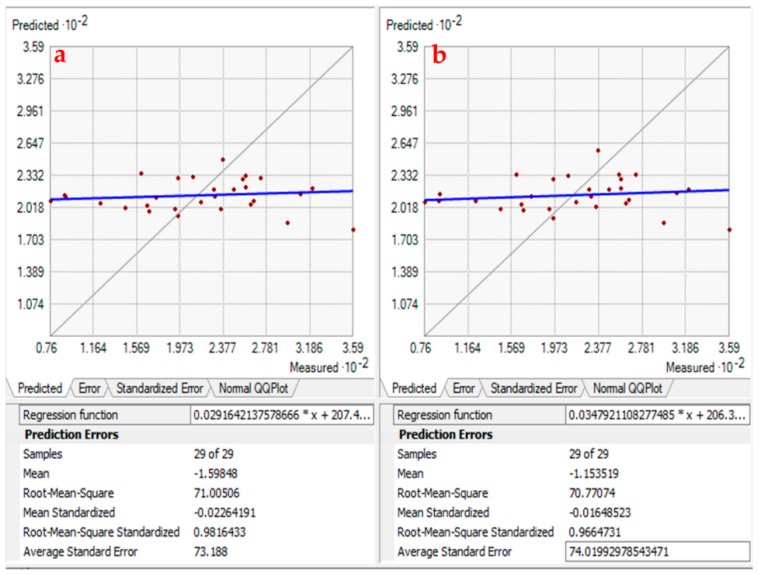
An example of Cross Validation Comparison. (**a**) Ordinary Kriging Spherical and (**b**) ordinary Kriging-Circular for Na element.

For the maps prediction, several kinds of semivariogram models were examined for each water quality parameter to obtain the preferable one, as seen in Figure 6 as an example. Predictive performances of the fitted models were checked on the basis of cross-validation tests. The values of mean error (ME), mean square error (MSE), root mean error (RMSE), average standard error (ASR) and root mean square standardized error (RMSSE) were estimated to ascertain the performance of the developed models. After conducting the cross-validation procedure, maps of kriged estimates were created that provided a visual representation of the distribution of the groundwater quality parameters in the Fw-Rh and Qa-Gu areas.

Kriging (Ordinary and Simple) interpolation method is the most suitable method in the studied areas. The value range of the better interpolation models were observed and reported in Table 3. If the RMSE is close to the ASE, the prediction errors were assessed correctly. If the RMSE is smaller than the ASE, then the variability of the predictions is overestimated; conversely, if the RMSE is greater than the ASE, then the variability of the predictions is underestimated. The same could be deduced from the RMSSE statistic. It should be close to one. If the RMSSE is greater than one, the variability of the predictions is underestimated; also, if it is minimal than one, the variability is overestimated. After generating the cross-validation procedure, estimated maps of kriging were created, which gives a visual representation of the distribution of the groundwater quality parameters.

**Figure 7:**
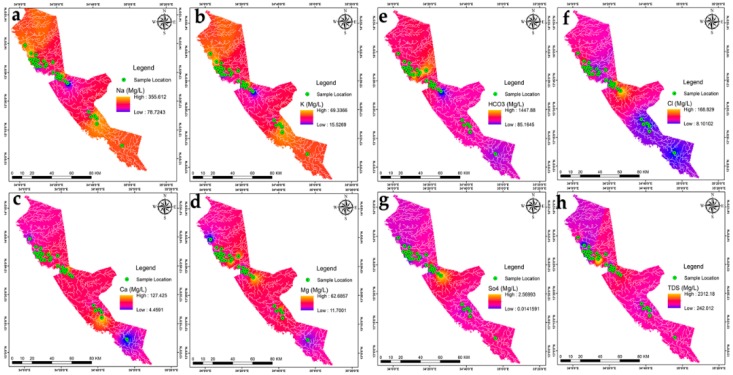
(**a**) Na, (**b**) K, (**c**) Ca, (**d**) Mg, (**e**) HCO_3_, (**f**) SO_4_, (**g**) Cl and, (**h**) TDS distributions in Fw-Rh area.

**Figure 8:**
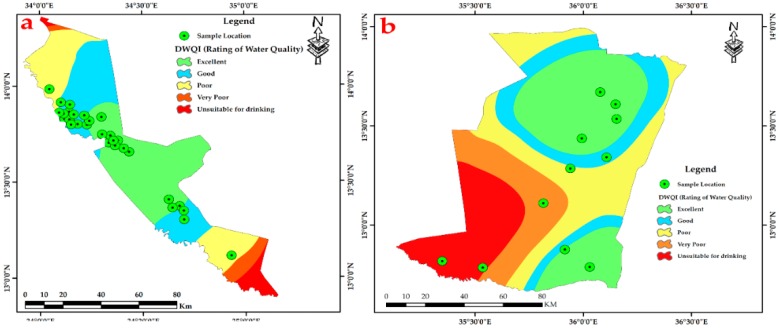
Spatial interpolation of Drinking Water Quality Index (DWQI). (**a**) Fw-Rh and (**b**) Qa-Qu localities Discussion.

The hydro-chemical of Fw-Rh area; the sodium concentration patterns in Figure 7a show similar trends to the potassium in Figure 7b with higher values in the northwest and southeast when decreasing in the central part. The distribution of calcium Figure 7c, ranges from 4.54 mg/L to 37.47 mg/L, it reflects relatively moderate values in the middle of Fw-Rh area. The distribution of magnesium Figure 7d, ranges from 11.66 mg/L to 34.28 mg/L. It also reflects the relatively moderate value in the middle of the area. The bicarbonate HCO_3_^−^ concentration Figure 7e, varies from 78.9 mg/L to 1450.00 mg/L, when the concentration of chloride Figure 7f, ranges from 8.10 mg/L to mg/L 172.50. The distribution of sulfate Figure 7g, ranges from 0.02 to 2.57, with a similar orientation as the TDS Figure 7h, both appear with higher values at the central part, moderate values in the southeast and low values in the northwest. The Fw-Ra consists most of the acidic rocks and Alluvium soil with clay layers in the project area, (i.e., granitic intrusions), thus, the origin of its most elements could be from weathering, hydro-chemical reactions and the solubility of minerals (i.e., The existence of potassium and sodium associated to the kaolin’s rich alunite (K, Na)Al_3_(SO_4_)_2_(OH)_6_] [36]); (Thirteen thematic layers of water quality parameters were used in ArcGIS environment to acquire the output of drinking water quality index DWQI maps for Fw-Rh Figure 8a, and Qa-Qu Figure 8b, localities. The water quality index was reclassified into five classes in order to characterize the quality of groundwater in the studied localities.

**Figure 9:**
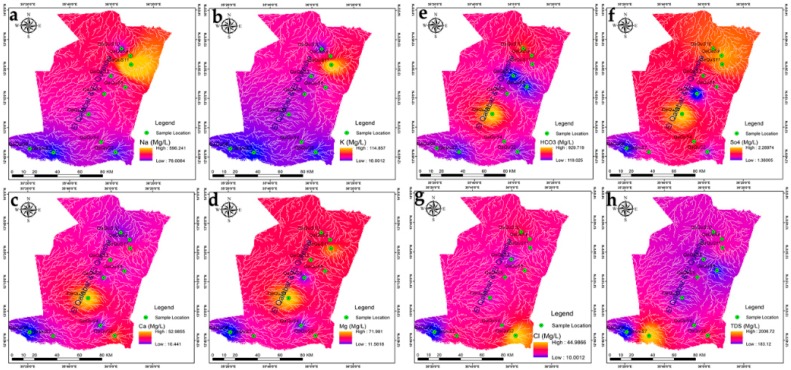
(**a**) Na, (**b**) K, (**c**) Ca, (**d**) Mg, (**e**) HCO_3_, (**f**) SO_4_, (**g**) Cl, and, (**h**) TDS distributions in Qa-Qu area.

**Figure 10:**
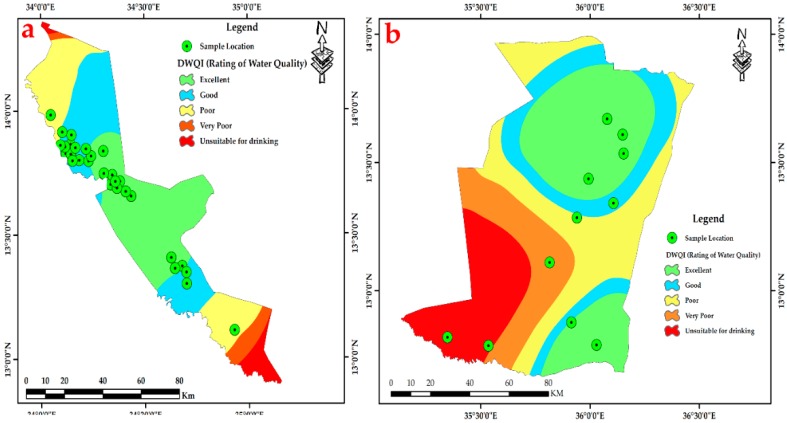
Spatial interpolation of Drinking Water Quality Index (DWQI). (**a**) Fw-Rh and (**b**) Qa-Qu localities discussion.

The hydro-chemical of Qa-Qu area; the sodium concentration in Figure 9a has similar trends as the potassium Figure 9b, with higher values in the east, middle values in the northwest to the north and low values in the southern part. The distribution of calcium in Figure 9c, ranges from 10.44 mg/L to 53.00 mg/L, the high values concentrated in the middle of Qa-Qu area, then decreases gradually to the north and south. The magnesium Figure 9d, ranges from 11.56 mg/L to 72.00 mg/L. It reflects random distribution values in the Qa-Qu area. The bicarbonate HCO_3_^−^ concentration Figure 9e, varies from 118.60 mg/L to 930.00 mg/L, when the concentration of chloride in Figure 9f, ranges from 10.00 mg/L to mg/L 45.00. The distribution of sulfate in Figure 9g, ranges from 1.36 to 2.29, with the highest values in the southeast and lowest values in the southwest. The TDS in Figure 9h, ranges from 183.00 mg/L to 2007.00 mg/L, appears with low to medium values at the central part then, increases to the southeasterly direction. The Qa-Qa area represented the most of Gedaref Formation, which is superimposed and intercalated by basaltic rocks (Oligocene) and substantially covered by the clay soils. Thus, the origin of most of its cations could be from chemical weathering, hydro-chemical reactions and the solubility of minerals (See thirteen thematic layers of water quality parameters used in ArcGIS environment to acquire the output of drinking water quality index DWQI maps for Fw-Rh Figure 10a, and Qa-Qu Figure 10b, localities. The water quality index was reclassified into five classes in order to characterize the quality of groundwater in the studied localities.

### 3.2. Correlation Matrix

The correlation matrix provides the assessment of the correlation coefficients “r” between groundwater quality elements. These coefficients are applied to suppress the strength of the linear relationship between the variables. It has been used to estimate both positive and negative correlations. The project area describes three examples of groundwater aquifers; (1) sandstone aquifer, dominant at Fw-Rh area; (11 Boreholes) and subdominant at Qa-Qu area; (5 Boreholes). (2) Alluvium aquifer, dominant at Fw-Rh area; (12 Boreholes) and only one borehole at Qa-Qu area. (3) Basaltic aquifer found as (five boreholes) at Qa-Qu area. (Table 4); Fw-Rh; reveal a strong positive correlation can be identified between Na^+^/K^+^ (r = 0.99), TDS/E.C (r = 0.99), E.C/Mg^+2^ (r = 0.55), TDS/Mg^+2^ (r = 0.53), and Ca^+2^/K^+^ (r = 0.51). It is found that NO_3_^−^ in most of the groundwater samples in this locality reflected strong correlations as: NO_3_^−^/TDS (r = 0.56), NO_3_^−^/E.C (r = 0.55), and NO_3_^−^/Ca^+2^ (r = 0.50).

(Qa-Qu); the magnesium has a strong positive correlation between most of the groundwater elements; Mg^+2^/Ca^+2^ (r = 0.75), Mg^+2^/SO_4_^−2^ (r = 0.73), Mg^+2^/HCO_3_^−^ (r = 0.54), Mg^+2^/K^+^ (r = 0.41), and Mg^+2^/Na^+^ (r = 0.40). Another positive correlation can be identified very strongly between; Na^+^/K^+^ (r = 0.99), HCO_3_^−^/SO_4_^−2^ (r = 0.70), NO_3_^−^/Fe^+2^ (r = 0.66), and pH/HCO_3_^−^ (r = 0.57). The strong negative correlation in (Qa-Qu) area, indicated as: Mg^+2^/NO_3_^−^ (r = −0.54), Cl^−^/Fe^+2^ (r = −0.46) pH/Na^+^ (r = −0.38), Mg^+2^/K^+^, (r = −0.38), and Ca^+2^/F^−^ (r = −0.35) (Table 5).

### 3.3. Groundwater Facies

**Figure 11:**
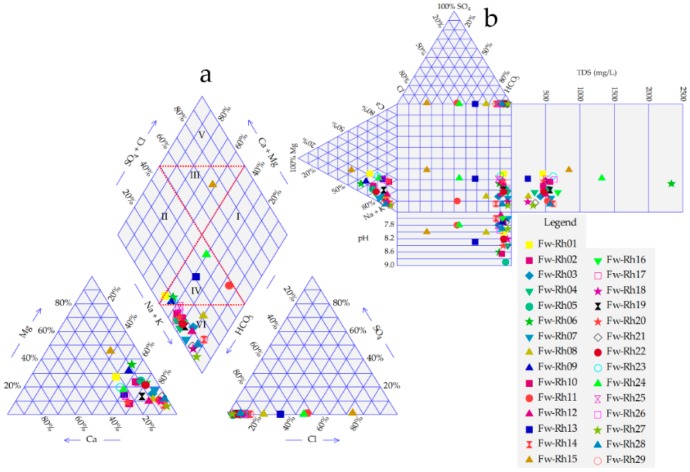
(**a**) Piper and (**b**) Durov diagrams of Fw-Rh area.

**Figure 12:**
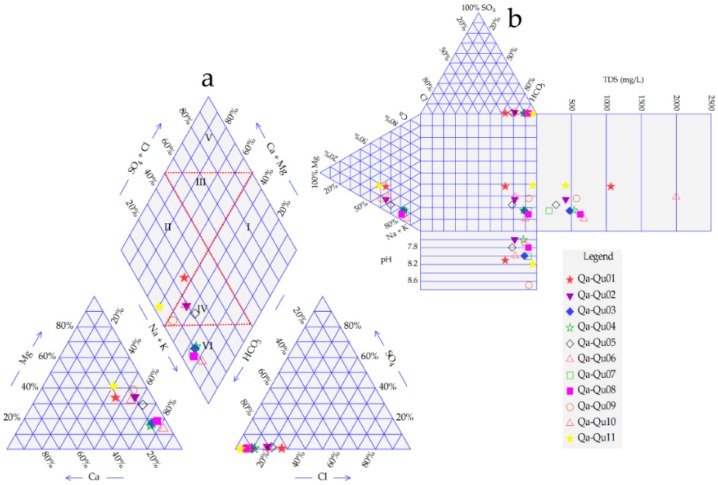
(**a**) Piper and (**b**) Durov diagrams of Qa-Qu area.

Groundwater facies were defined by applying a Piper plot and Durov diagrams as seen in Figure 11 and Figure 12. The descriptions reveal that the area consists of eight groups of groundwater types Table 6, Na-Mg-HCO_3_, Na-HCO_3_, Na-Ca-HCO_3_, Na-Ca-Mg-HCO_3_, Mg-Na-Ca-Cl, Mg-Na-HCO_3_, Na-Ca-Mg-HCO_3_-Cl, and Na-Mg-Ca-HCO_3_. The analytical results achieved from the samples when plotted on Piper’s plot, explained that the alkalis (Na^+^, K^+^), appear considerably over the alkaline elements (Ca^+2^, Mg^+2^), and the weak acidic (HCO_3_^−^) appear considerably over strong acidic anions (Cl^−^ & SO_4_^−2^). Moreover, Piper diagram matched 40% of the samples, under Na-Mg-HCO_3_ group and 35% under Na-HCO_3_ type.

According to the plotting from the Durov diagram, most of the elements of water plotted within the HCO_3_·Na zone, except some other samples that were fell in HCO_3_ Cl–Na, SO_4_·Cl·HCO_3_–Na, or HCO_3_·Cl–Na·Mg types.

### 3.4. Drinking Water Quality Index (DWQI)

To gain a comprehensive representation of the quality of the drinking groundwater, drinking water quality index (DWQI) is one of the useful tools. It supplies a single amount to a state’s overall water quality at a specific location and time, based on a number of water quality parameters. DWQI in Table 7, was calculated by adopting weighted arithmetical index method considering thirteen water quality parameters (pH, TDS, Ca^+2^, Mg^+2^, Na^+^, K^+^, Fe^+2^, Cl^−^, HCO_3_^−^, SO_4_^−2^, F^−^, NO_3_^−^, and E.C) in order to assess the degree of groundwater contamination and suitability.

**Figure 13:**
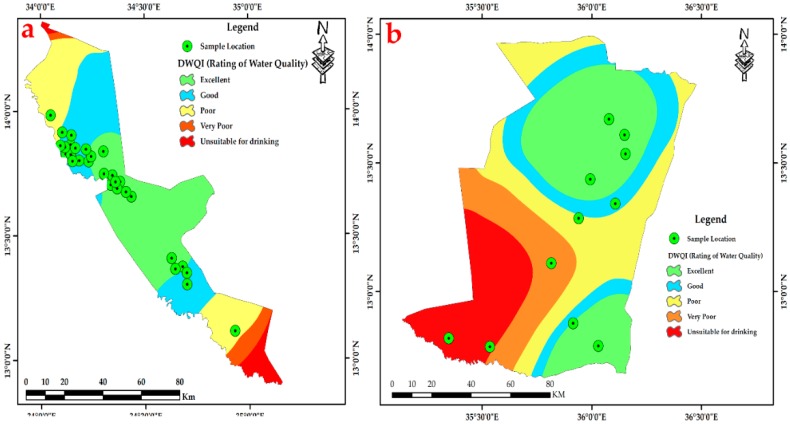
Spatial interpolation of DWQI, (**a**) Fw-Rh and (**b**) Qa-Qu localities.

Thirteen thematic layers of water quality parameters were used in the ArcGIS environment to acquire the output of drinking water quality index DWQI maps for Fw-Rh Figure 13a, and Qa-Qu Figure 13b, localities. The water quality index was reclassified into five classes in order to characterize the quality of groundwater in the studied localities.

## 4. Discussion

In this study, most of the boreholes were recently drilled (2015–2017), no other boreholes were available. Due to the few observation points, limited previous investigations and few hydrogeological data, using geospatial distributions, GIS and DWQI provide support in groundwater studies. As far as we know, no other study was conducted using the techniques in Fw-Rh and Qa-Qu areas.

**Figure 14:**
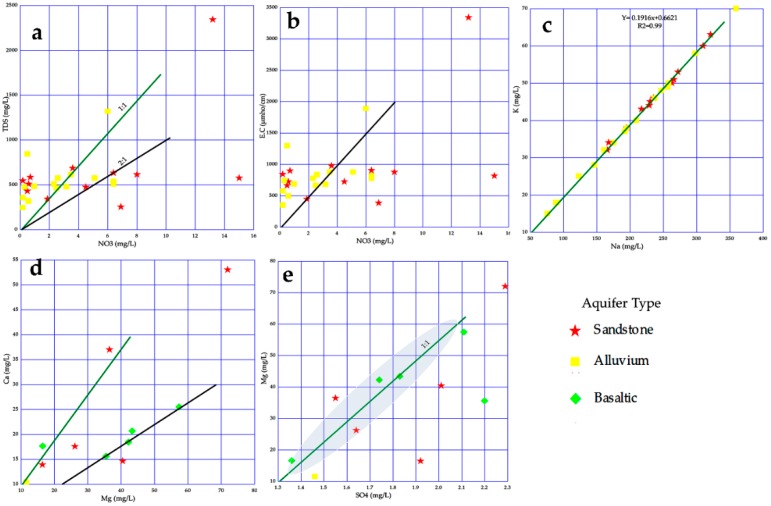
Bivariate diagrams of ionic relations in groundwater of different aquifers.

**Figure 15:**
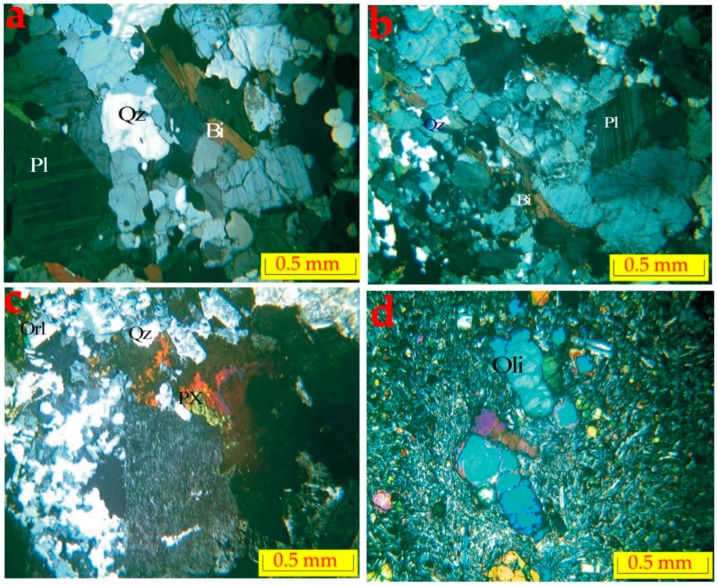
Selective rock thin sections: (**a**) Granite, (**b**) Migmatite gneiss, (**c**) Quartz Syenite, and Oligocene basalt (Qtz = quartz, Pl = plagioclase, Bi = Biotite, Orl = orthoclase, Px = pyroxene, & Oli = olivine).

The chemical composition and elements concentration of groundwater, are related to the rocks lithology and time residence of the water in the aquifers. To identify the effects of the reaction between the groundwater and the (geological units) aquifer, the bivariate diagrams were applied to explain the chemical changes in ionic concentrations in the host rocks and groundwater. The reaction between water and the surrounding surface/soil from agricultural fields can change groundwater chemistry. The bivariate diagram of NO_3_ vs. TDS and E.C, Figure 14a,b, records that six samples of alluvium aquifer and two samples of sandstone aquifer, plots along the 1:1 aquiline, show the highest correlation among TDS/NO_3_. The appearance of NO_3_ associated with the fertilizers activities in the agricultural farms [37,38,39]. The elements Na^+^ versus K^+^ Figure 14c, at both sandstone and alluvium aquifers, reflects a linear relationship at (r = 0.99) suggesting the reactions of water with sodium feldspar (Albite) and potassium feldspar (Orthoclase) in equations five and six respectively. The Mg^+2^ strongly correlated with Ca^+2^ and SO_4_^−2^ Figure 14d,e, especially in the basaltic aquifer and some other boreholes in the alluvium locations, show a high response of water with a group of minerals (i.e., Pyroxene, Olivine and Biotite) in equations seven, eight, and nine respectively. For the better understanding of geological units in research areas, the thin sections of rock samples have been generated and studied to identify the main mineral composition for each rock sample, as seen in Figure 15. With this ability, the rock mineral contents have been determined much better.

The main mechanism for the dissolution of rock minerals that releases the element such as: (Ca, Mg, Na, K and HCO_3_); into the groundwater, have been indicated in the following reactions:

(4) $$\left(\mathrm{Pl}\right)\mathrm{Anorthite}:{\;\mathrm{CaAl}}_{2}{\mathrm{Si}}_{2}O_{3}+2{\mathrm{CO}}_{3}+H_{2}O\;\to {\;\mathrm{Al}}_{2}{\mathrm{Si}}_{2}O_{5}{\left(\mathrm{OH}\right)}_{4}+{\mathrm{Ca}}_{2}^{+}+2{\mathrm{HCO}}_{3}$$

(5) $$\left(\mathrm{Pl}\right)\mathrm{Albite}:2{\mathrm{NaAl}}_{3}O_{8}+9H_{2}O+2H^{+}\;\to {\mathrm{Al}}_{2}{\mathrm{Si}}_{2}O_{5}{\left(\mathrm{OH}\right)}_{4}+4H_{4}{\mathrm{SiO}}_{4}+2{\mathrm{Na}}^{+}$$

(6) $$\left(\mathrm{Orl}\right)\;\mathrm{Orthoclase}:2{\mathrm{KAlSi}}_{3}O_{8}+11H_{2}O\;\to {\mathrm{Si}}_{2}O_{5}{\mathrm{Al}}_{2}{\left(\mathrm{OH}\right)}_{4}+2K^{+}+2{\mathrm{OH}}^{-}$$

(7) $$\left(\mathrm{Px}\right)\mathrm{Pyroxene}:\;\mathrm{CaMg}\left({\mathrm{Si}}_{2}O_{6}\right)+4{\mathrm{CO}}_{2}+6H_{2}O\;\to {\mathrm{Ca}}^{2}{}^{+}+{\mathrm{Mg}}^{2}{}^{+}\;4{\mathrm{HCO}}_{3}^{-}+2\mathrm{Si}{\left(\mathrm{OH}\right)}_{4}$$

(8) $$\begin{array}{c} \left(\mathrm{Oli}\right)\mathrm{Olivine}\left(\mathrm{Fe},{\mathrm{Mg}}^{2+}\right)SiO_{4}+4H_{2}{\mathrm{CO}}_{3}\;\to 2\left(\mathrm{Fe},{\mathrm{Mg}}^{2+}\right)+H_{4}\mathrm{Si}O_{4}+4{\mathrm{HCO}}_{3}^{-} \end{array}$$

(9) $$\left(\mathrm{Bi}\right)\;\mathrm{Biotite}:\;K{\left(\mathrm{Mg},\;\mathrm{Fe}\right)}_{3}\left({\mathrm{AlSi}}_{3}O_{10}\right)\left(F,{\mathrm{OH}}_{2}\right)+5H_{2}O+4{\mathrm{CO}}_{2})\;\to K+\mathrm{Mg}+\mathrm{Fe}{\left(\mathrm{OH}\right)}_{3}+4{\mathrm{HCO}}_{3}+H+2F$$

## 5. Conclusions

This study explains the geospatial distribution, adopting statistical methods with GIS to characteristics and mapped the groundwater quality in the different hydrogeological units such as sandstone, alluvium, and basaltic aquifers, which are located in eastern Sudan (the southwestern part of Gedaref State). Forty water boreholes samples from different locations were collected, analyzed and estimated.

Aqua Chem v.2014.2 software has been used for groundwater quality elements analysis, while ArcGIS software was chosen for the interpretation and spatial mapping, so that groundwater quality estimation studies have been completed successfully. This study envisions the significance of graphical illustrations, i.e., Piper, Bivariate, Dendrogram, and Durov diagrams plot, to determine variation in hydro-chemical facies and to understand the evolution of hydro-chemical processes in Qa-Qu and Fw-Rh areas.

The hydrogeochemical evaluation outcomes and distribution of groundwater cations (Na^+^, Ca^+2^, K^+^, Mg^+2^) and anions (HCO_3_^−^, Cl^−^, SO_4_^−2^, F^−^) in both the Qa-Qu and Fw-Rh areas, shows that the groundwater is chemically affected by aquifer lithology. According to the plotting from the Durov diagram, most of the elements of water plotted within the HCO_3_·Na zone, except some other samples that fell in HCO_3_ Cl–Na, SO_4_·Cl·HCO_3_–Na, or HCO_3_·Cl–Na Mg types. With the exclusion of a few elements, the quality of groundwater is mostly suitable for drinking purposes and other domestic uses. The groundwater in this project is controlled by sodium and bicarbonate ions, which define the composition of the water type to be Na HCO_3_. According to this investigation, three potential aquifers (sandstone, alluvium, and basalt); have been identified in the research areas.

The DWQI was used to determine the groundwater quality and its suitability for drinking purposes. According to this investigation, 20% of groundwater samples represent “excellent water”, 50% indicate “good water”, 15% represent “poor water”, 7.5% shows “very poor water”, and 7.5% appear as “unsuitable for drinking”. The drinking water quality index that was produced for this study reveals that the northwest and southeast parts of Fw-Rh and the southwest part of Qa-Qu locations has the poorest water quality, which is classified as “unsuitable for drinking”.

It should be noted, that the actual variations in spatial interpolations, can considerably diverge from the values predicted by spatial interpolation, it may lead to probable limitations of Kriging especially when data is scarce and unequally distributed. Thus, it is essential to know the number of data locations and the geographical extent of the region containing those data locations. In this case, one of the crucial steps is estimating the variogram model, which is more difficult with a small number of data locations. In this study, the transformations (Log & BoxCox), have been used to make the data normally distributed and satisfy the assumption of equal variability for the data. Several types of semivariogram models were tested in Table 3, for all water quality parameters to achieve more reliable results.

## Figures and Tables

**Table 1:** Hydrogeological data of forty wells drilled in the study area.

**Aquifer Type**	**Well Depth (m)**	**S.W.L (M)**	**Elevation (m)**	**Water Table (m)**	**Well Name**
Alluvium	23	16	425	409	Ellewatah3
Sandstone	27	16	424	408	Abu Kalbo
Sandstone	64	37	423	386	Um Rakuba
Sandstone	48	27	425	398	Um Tireaza
Sandstone	21	11	444	432	Um Tireaza 2
Sandstone	60	13	554	505	Macancana
Sandstone	48	11.94	426	414	Wd Margi
Sandstone	21	11	446	435	Mohamed Ali
Alluvium	14	7	424	417	Um Gazaz
Sandstone	64	12	425	413	Wad Elkarar1
Alluvium	27	19	424	405	Eldar Elbeida
Sandstone	42	13	655	634	Ellewatah2
Alluvium	27	16	486	470	Halaly5
Alluvium	30	14	427	413	Wad Elwosta
Alluvium	57	34	424	383	Wad Elkarar2
Basaltic	42	24	423	410	Hilat Ali
Alluvium	22.5	12	428	416	Abu Saeed
Alluvium	28	13	427	414	Elyas
Alluvium	26	12	648	634	Elmeageerah
Sandstone	25	7	598	591	Dora
**Well Code**	**Aquifer Type**	**Well Depth (m)**	**S.W.L (M)**	**Elevation (m)**	**Water Table (m)**
Fw-Rh21	Alluvium	34	14	447	433
Fw-Rh22	Alluvium	18	10	444	434
Fw-Rh23	Alluvium	57	41	433	399
Fw-Rh24	Alluvium	26	18	428	410
Fw-Rh25	Alluvium	21	14	422	411
Fw-Rh26	Basaltic	15	8	658	650
Fw-Rh27	Alluvium	24	10	428	418
Fw-Rh28	Alluvium	21	8	431	423
Fw-Rh29	Alluvium	23	16	425	409
Qa-Qu01	Sandstone	53	32	627	595
Qa-Qu02	Sandstone	60	49	426	413
Qa-Qu03	Alluvium	21	12	422	411
Qa-Qu04	Basaltic	42	21	631	607
Qa-Qu05	Basaltic	26	14	454	442
Qa-Qu06	Sandstone	43	35	500	465
Qa-Qu07	Alluvium	27	16	428	412
Qa-Qu08	Sandstone	21	11	431	417
Qa-Qu09	Sandstone	57	42	635	593
Qa-Qu10	Sandstone	31	21	447	426
Qa-Qu11	Basaltic	30	9	650	641

**Table 2:** Descriptive statistical analysis result of water samples (N = 40)**.**

	Fw-Rh (N = 29)					Qa-Qu (N = 11)			
Variable	WHO	Minimum	Maximum	Mean	Std. D.	Minimum	Maximum	Mean	Std. D.
pH	7.0–8.0	7.50	8.90	8.03	0.35	7.60	8.70	7.96	0.32
TDS (mg/L)	1000	242.00	2340.00	601.72	399.60	183.00	2007.00	663.18	502.41
Ca	75	4.45	130.00	37.47	30.06	10.44	53.00	22.21	12.43
Mg	30	11.66	120.53	34.28	23.86	11.56	72.00	36.20	18.23
Na	200	76.00	359.00	214.28	69.87	76.00	597.00	167.18	150.29
K	-	15.00	70.00	41.72	13.40	16.00	115.00	33.36	28.68
Cl	250	8.10	172.50	35.90	37.69	10.00	45.00	21.39	8.96
HCO_3_	-	78.90	1450.00	499.35	311.78	118.60	930.00	355.40	247.40
SO_4_	250	0.02	2.57	0.74	0.70	1.36	2.29	1.83	0.31
F	0.5–1	0.01	2.56	0.55	0.55	0.02	2.56	0.65	0.70
NO_3_	50	0.20	15.00	3.88	3.96	1.50	11.10	5.05	3.11
Fe	0.03	0.01	0.80	0.15	0.22	0.01	1.04	0.19	0.31
E.C (µ.S/cm)	-	345.00	3342.00	888.23	570.54	281.53	9692.00	1777.97	2721.19

(WHO)= The world health organization standard.

**Table 3:** Characteristics parameters of variogram models.

Area	Groundwater Parameters	Kriging Type	Transformation	Best Fitted Model	ME	RMSE	ASE	MSE	RMSSE
Fw-Rh	Na	Simple	None	Spherical	−1.5985	71.0051	73.1880	−0.0226	0.9816
	K	Ordinary	None	Spherical	−0.2818	13.6108	14.0445	−0.0207	0.9817
	Ca	Ordinary	Log	Circular	0.6579	32.1486	30.1062	0.0155	1.0624
	Mg	Ordinary	Log	Gaussian	0.5210	18.9853	22.6372	−0.0224	0.8808
	HCO_3_	Ordinary	BoxCox	Stable	19.5080	328.5189	367.5360	0.0498	0.9083
	Cl	Ordinary	Log	Circular	−0.4151	38.0299	37.4075	−0.0077	1.0432
	SO_4_	Ordinary	BoxCox	Stable	0.0593	0.6693	0.6351	0.0892	1.0814
	TDS	Ordinary	BoxCox	Stable	12.4257	434.6693	453.4310	0.0294	1.0040
Qa-Qu	Na	Simple	None	Stable	10.9458	143.4509	156.6227	0.0688	0.8907
	K	Simple	Log	Spherical	−0.1683	27.1142	19.2757	−0.0102	1.4215
	Ca	Simple	BoxCox	Stable	−0.3761	14.1042	11.5697	−0.0176	1.1645
	Mg	Ordinary	Log	Circular	1.6593	20.6180	23.8640	−0.0615	1.0218
	HCO3	Ordinary	BoxCox	Stable	15.3778	261.7170	258.9817	0.0556	0.9939
	Cl	Ordinary	Log	Stable	−0.3736	9.6335	7.1223	−0.1165	1.2648
	SO4	Ordinary	None	Spherical	0.0251	0.3209	0.3193	0.0654	1.0026
	TDS	Simple	BoxCox	Gaussian	6.7649	614.1923	453.6139	−0.2829	1.8464

(ME)= Values of mean error, (RMSE)= Root mean error, (ASE)= Average standard error, (MSE)= Mean square error, (RMSSE)= Root mean square standardized error.

**Table 4:** Correlation matrix analysis result of the groundwater quality parameters in Fw-Rh area.

Variables	pH	TDS	Ca	Mg	Na	K	Cl	HCO_3_	SO_4_	F	NO_3_	Fe	E.C
pH	**1**												
TDS	**0.20**	**1**											
Ca	0.11	**0.26**	**1**										
Mg	0.10	**0.53**	**0.31**	**1**									
Na	**−0.30**	0.02	**0.23**	0.07	**1**								
K	**−0.31**	0.02	**0.23**	0.08	**0.99**	**1**							
Cl	**−0.14**	**0.33**	**0.51**	**0.49**	**0.13**	**0.15**	**1**						
HCO_3_	−0.11	**−0.16**	−0.07	0.10	−0.01	0.00	−0.10	**1**					
SO_4_	0.10	0.05	−0.08	0.01	**−0.17**	**−0.18**	0.06	**−0.34**	**1**				
F	**−0.26**	**−0.17**	**−0.35**	**−0.21**	**0.14**	**0.13**	**−0.18**	0.09	**−0.26**	**1**			
NO_3_	**0.25**	**0.56**	**0.49**	**0.32**	**0.24**	**0.24**	**0.16**	**−0.19**	0.03	**−0.21**	**1**		
Fe	**−0.15**	**−0.14**	**−0.15**	**−0.41**	−0.02	−0.02	−0.09	**−0.26**	**−0.17**	0.13	−0.06	**1**	
E.C	**0.19**	**0.99**	**0.24**	**0.55**	0.00	0.00	**0.34**	**−0.16**	0.05	**−0.16**	**0.55**	**−0.15**	**1**

Values in bold are different from 0 with a significance level alpha = 0.5.

**Table 5:** Correlation matrix analysis result of the groundwater quality parameters in Qa-Qu area.

Variables	pH	TDS	Ca	Mg	Na	K	Cl	HCO_3_	SO_4_	F	NO_3_	Fe	E.C
**pH**	**1**												
**TDS**	0.09	**1**											
**Ca**	0.15	0.05	**1**										
**Mg**	0.13	−0.09	**0.75**	**1**									
**Na**	**−0.38**	−0.09	0.04	**0.40**	**1**								
**K**	**−0.38**	−0.09	0.04	**0.41**	**0.99**	**1**							
**Cl**	**0.33**	**0.45**	**0.29**	0.06	−0.19	−0.19	**1**						
**HCO_3_**	**0.57**	−0.21	**0.52**	**0.54**	0.00	0.01	−0.07	**1**					
**SO_4_**	**0.24**	−0.16	**0.37**	**0.73**	**0.43**	**0.44**	−0.15	**0.70**	**1**				
**F**	**−0.27**	−0.22	**−0.35**	−0.18	−0.09	−0.09	−0.16	**−0.42**	**−0.30**	**1**			
**NO_3_**	**−0.26**	0.06	−0.19	**−0.54**	−0.15	−0.15	**−0.34**	−0.08	**−0.50**	−0.16	**1**		
**Fe**	0.18	−0.16	−0.05	**−0.26**	**−0.29**	**−0.29**	**−0.46**	0.08	**−0.27**	−0.11	**0.66**	**1**	
**E.C**	−0.13	**0.24**	−0.16	−0.04	0.14	0.16	0.13	0.17	**0.34**	**−0.27**	0.15	**−0.23**	**1**

Values in bold are different from 0 with a significance level alpha = 0.5.

**Table 6:** Groundwater facies distribution in the study area.

Water Type	Fw-Rh	Qa-Qu	Water Type %
Na-Mg-HCO_3_	10	6	16 (40%)
Na-HCO_3_	11	3	14 (35%)
Na-Ca-Mg-HCO_3_	3	0	3 (7.5%)
Na-Ca-HCO_3_	3	0	3 (7.5%)
Mg-Na-Ca-Cl	1	0	1 (2.5%)
Mg-Na-HCO_3_	0	1	1 (2.5%)
Na-Ca-Mg-HCO_3_-Cl	1	0	1 (2.5%)
Na-Mg-Ca-HCO_3_	0	1	1 (2.5%)

**Table 7:** DWQI for groundwater samples in (Fw-Rh-Qa-Qu) area.

DWQI Value	Rating of Water Quality	Percent %	Fw-Rh Area	Qa-Qu Area
**0–25**	Excellent	8 (20%)	5	3
**25–50**	Good	20 (50%)	17	3
**51–100**	Poor	6 (15%)	3	3
**101–200**	Very Poor	3 (7.5%)	2	1
**>>200**	Unsuitable for drinking	3 (7.5%)	2	1
